# Transfemoral Bridging Stent-Graft Delivery in Zone 0 Endovascular Arch Repair With Triple-Fenestrated Endograft

**DOI:** 10.1093/icvts/ivaf209

**Published:** 2025-09-16

**Authors:** Hiroaki Kaneyama, Kenichi Hashizume, Toshiaki Yagami, Kiyoshi Koizumi, Koki Ikebata, Takashi Hashimoto, Masayoshi Waga, Hideyuki Shimizu

**Affiliations:** Department of Cardiovascular Surgery, Keio University School of Medicine, Tokyo, 1608582, Japan; Department of Cardiovascular Surgery, Red Cross Ashikaga Hospital, Tochigi, 3260843, Japan; Department of Cardiovascular Surgery, Keio University School of Medicine, Tokyo, 1608582, Japan; Department of Cardiovascular Surgery, Saiseikai Utsunomiya Hospital, Tochigi, 3210974, Japan; Department of Radiology, Saiseikai Utsunomiya Hospital, Tochigi, 3210974, Japan; Department of Cardiovascular Surgery, Red Cross Ashikaga Hospital, Tochigi, 3260843, Japan; Department of Cardiovascular Surgery, Red Cross Ashikaga Hospital, Tochigi, 3260843, Japan; Department of Cardiovascular Surgery, Red Cross Ashikaga Hospital, Tochigi, 3260843, Japan; Department of Cardiovascular Surgery, Red Cross Ashikaga Hospital, Tochigi, 3260843, Japan; Department of Cardiovascular Surgery, Keio University School of Medicine, Tokyo, 1608582, Japan

**Keywords:** endovascular aortic arch repair, physician-modified endograft, fenestration, Zone 0 landing

## Abstract

Zone 0 thoracic endovascular aortic repair (TEVAR) remains technically demanding because of limited proximal landing zones and the need to preserve all supra-aortic branches. Conventional strategies—including branched endografts, chimney or snorkel techniques, and hybrid repairs—have been associated with increased risks of stroke, retrograde type A dissection, and perioperative mortality.

We describe a technique using a physician-modified triple-fenestrated endograft with transfemoral delivery of all bridging covered stents (BCSs) to the brachiocephalic, left common carotid, and left subclavian arteries. Cervical and brachial access was used solely for angiography and minimal catheter manipulation, thereby aiming to reduce cerebral embolization risk.

A 71-year-old man with a history of coronary artery bypass surgery and reduced left ventricular ejection fraction (31%) presented with fever. Imaging revealed a dissecting aortic aneurysm confined to the arch. Blood cultures were positive for methicillin-susceptible *Staphylococcus aureus*. After intravenous antibiotic therapy, cultures became negative; however, the aneurysm enlarged. Given the high surgical risk, TEVAR was selected. All BCSs were delivered transfemorally without complications. Postoperative and 1-year follow-up imaging showed no endoleak and a reduction in aneurysm size.

This approach may offer a less invasive and embolic risk-reducing option for managing arch pathology in high-risk patients.

## INTRODUCTION

Thoracic endovascular aortic repair (TEVAR) involving Zone 0 remains technically demanding because of limited proximal landing zones and the necessity of preserving all supra-aortic branches. Conventional solutions—including branched endografts, chimney or snorkel techniques, and hybrid repair with extra-anatomic bypasses—have been associated with increased rates of stroke, retrograde type A dissection (RTAD), and perioperative mortality.^1^^,^^2^ Physician-modified endografts (PMEGs) have emerged as a more flexible alternative. However, they often require complex alignment of multiple fenestrations with supra-aortic branches, particularly in the ascending aorta, and typically involve carotid or brachial access for bridging stent delivery.^3^ These maneuvers may increase the risk of cerebral embolism.^4^

We report a case using a triple-fenestrated PMEG with transfemoral delivery of all bridging covered stents (BCSs), designed to reduce embolic events by limiting cervical manipulation to the minimum necessary.

## PATIENT

**Figure 1.**
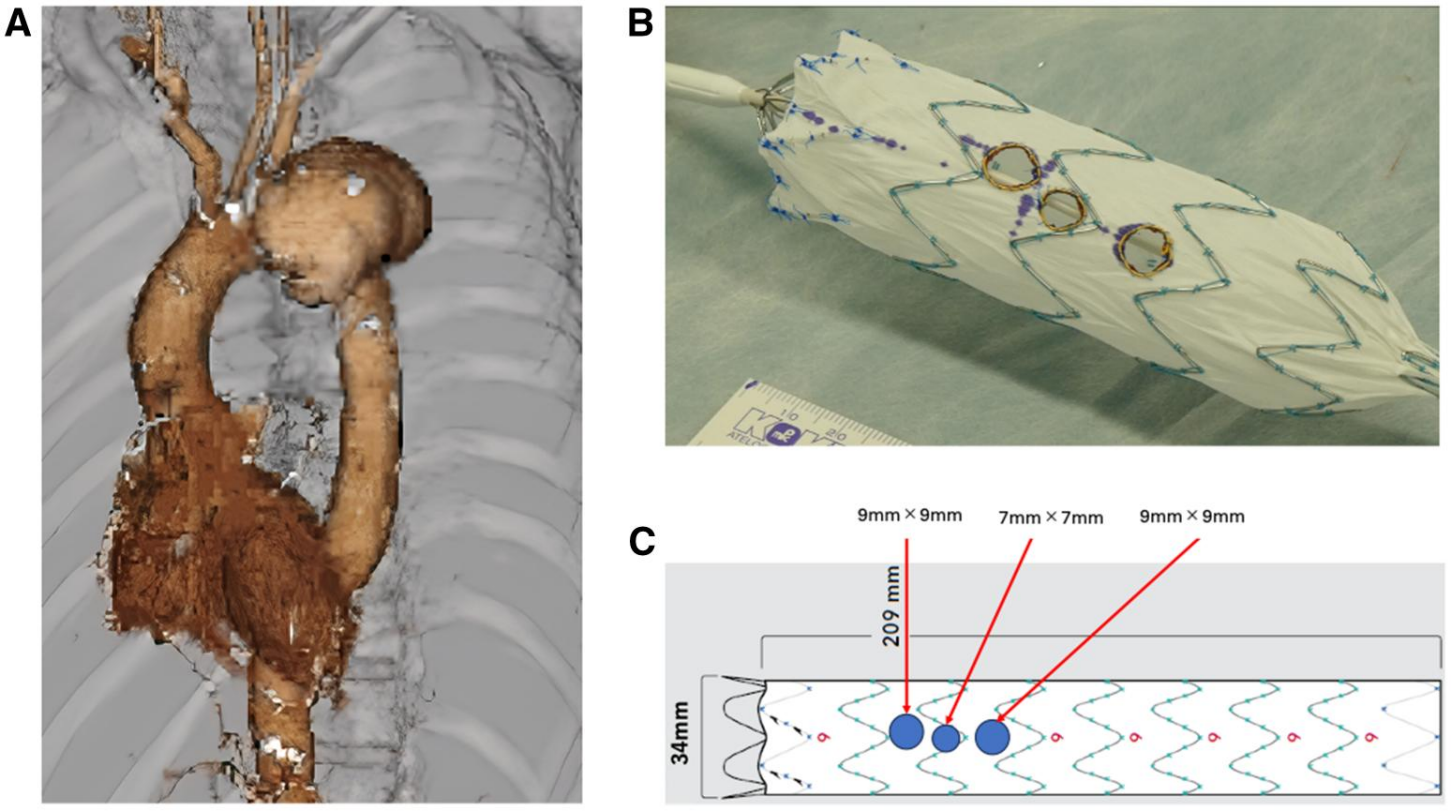
Preoperative Imaging and Fenestrated Stent-Graft Preparation. (A) Preoperative aortic computed tomography angiogram showing a dissecting aortic aneurysm in the arch. (B, C) Three fenestrations (brachiocephalic artery: 9 × 9 mm, left common carotid artery: 7 × 7 mm, and left subclavian artery: 9 × 9 mm) were created on the stent-graft using high-temperature cautery

A 71-year-old man with a history of coronary artery bypass grafting (CABG) presented to another hospital with fever. Computed tomography (CT) revealed a dissecting aortic aneurysm confined to the arch, which had not been present on a CT performed 4 months earlier, and a pseudoaneurysm of the proximal left anterior descending artery (**Supplement 1A**). He was referred to our hospital for further evaluation. Blood cultures were positive for methicillin-susceptible *Staphylococcus aureus*. After intravenous antibiotic therapy, the pseudoaneurysm regressed (**Supplement 1B**). However, the aortic aneurysm enlarged by 15 mm over 3 months (**Figure 1A**). A mycotic pseudoaneurysm of the arch was considered possible. Redo open surgery was deemed prohibitively high risk because of the patient’s prior CABG and reduced ejection fraction (31%). With blood cultures negative after antibiotic therapy, TEVAR was selected as the most feasible treatment option.

## SURGICAL TECHNIQUE

### Stent-graft processing

The Zenith Alpha Thoracic Endovascular Graft (Cook Medical, Bloomington, IN, United States; 34-mm central diameter [6.25% oversizing] × 34-mm peripheral diameter × 209-mm length) was partially withdrawn from its sheath on the back table to expose approximately 5 proximal stent segments. The proximal fixation barbs were removed. The positions of the cervical branches were precisely measured based on preoperative CT. Three fenestrations were created in the stent graft (SG) using high-temperature cautery: 9 × 9 mm, 7 × 7 mm, and 9 × 9 mm for the brachiocephalic artery (BCA), left common carotid artery (LCCA), and left subclavian artery (LSCA), respectively (**Figure 1B and C**). Each fenestration was 3-4 mm smaller than the BCS, and radiopaque markers were sewn around the circumference. The SG was reloaded into the original sheath using 5-mm polyester tape. The diameters of the BCSs were selected to be approximately 3-4 mm larger than the native cervical branch diameters.

### Implantation

Under general anaesthesia, the left femoral artery (LFA) and LCCA were surgically exposed. A 6 Fr Glidesheath Slender (Terumo, Tokyo, Japan) was inserted percutaneously into both brachial arteries and directly into the LCCA, and a temporary pacing wire was positioned in the right ventricle via the right femoral vein.

Initial aortography was performed using a PIG catheter advanced from the left brachial artery (LBA). The base SG was deployed with at least 3 stent segments landing distally, ensuring that the proximal end did not obstruct the LSCA.

**Figure 2.**
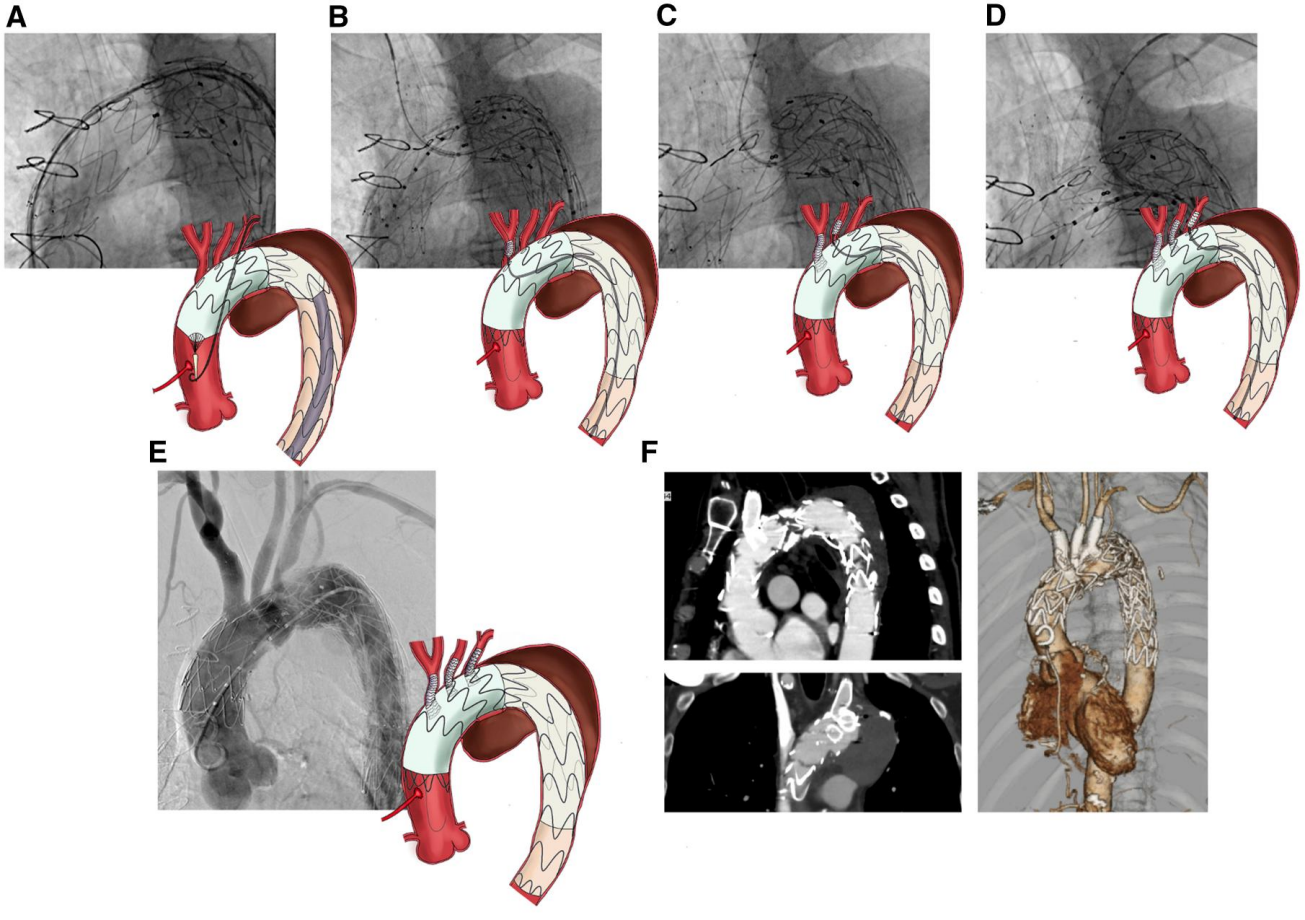
Stepwise implantation using transfemoral bridging stent-graft delivery with postoperative imaging. (A) Fenestrated physician-modified endograft (f-PMEG) was deployed with the radiopaque marker positioned slightly more centrally than the cervical branch. (B) A pull-through wire was inserted between the right brachial artery and left femoral artery, and a bridging covered stent (VIABAHN, 13 mm × 5 cm) was deployed. (C) A pull-through wire was inserted between the left common carotid artery and left femoral artery, and a bridging covered stent (VIABAHN, 10 mm × 5 cm) was deployed. (D) A pull-through wire was inserted between the left brachial artery and left femoral artery, and a bridging covered stent (VIABAHN, 13 mm × 5 cm) was deployed. (E) Aortography showing leakage of contrast medium into the aneurysm (a type 4 endoleak). (F) Follow-up computed tomography (CT) on postoperative day 6 showing no endoleaks or abnormalities of the supra-aortic branches

The triple-fenestrated PMEG was advanced over a Lunderquist guidewire. Aortography confirmed the positions of the supra-aortic branches. The PMEG was deployed under rapid pacing at 150 bpm, and the radiopaque fenestration markers were aligned slightly proximal to the branch origins (**Figure 2A**).

Three VIABAHN stents (W. L. Gore, Flagstaff, AZ, United States) were deployed as BCSs, all via transfemoral access:

BCA: A pull-through wire was established between the right brachial artery and LFA; a 13 × 50 mm VIABAHN was deployed (**Figure 2B**).
LCCA: A pull-through wire from the LCCA to LFA was used to deploy a 10 × 50 mm VIABAHN (**Figure 2C**).
LSCA: A 13 × 50 mm VIABAHN was introduced from the LFA using a pull-through wire to the LBA. During delivery, the stent tip became lodged at the edge of the fenestration. A contrast catheter inserted from the LBA was used to align the stent tip coaxially with the fenestration, eliminating the step-off and enabling smooth advancement (**Figure 2D**).

Final aortography revealed minimal contrast leakage into the aneurysm sac, consistent with a type IV endoleak, which was considered clinically insignificant (**Figure 2E**). The total procedure time was 206 min, with 133 min of fluoroscopy and 316 mL of contrast used. The patient had an uneventful postoperative course. CT on postoperative day 6 confirmed the absence of endoleaks (**Figure 2F**). Follow-up CT at 1 year likewise demonstrated no endoleaks and a reduction in aneurysm size (**Supplement 2**, **Video 1**). Antibiotic therapy was discontinued at 12 months because of stable inflammatory markers.

## DISCUSSION

Stroke remains a major concern in Zone 0 TEVAR, where risk is multifactorial, involving cerebral ischaemia time, device manipulation, and embolization of unstable plaques. In our case, all BCSs were delivered transfemorally, minimizing catheter manipulation within the supra-aortic branches. Consequently, only a small-caliber sheath was required from the cervical side, reducing stress on the branch ostia compared with conventional retrograde approaches. Moreover, by using femoral access, BCSs could be deployed in a distal-to-proximal direction—from the distal branch towards its aortic origin—which may reduce the risk of cerebral embolization caused by an embolic shower into the cerebral circulation. Although conceptually attractive, this benefit remains unproven.

Achieving accurate fenestration alignment with the supra-aortic branches is technically challenging. The Zenith Alpha device, with its pre-curved design, generally maintains orientation^5^; however, twisting during loading may still cause misalignment. In such cases, the device should be withdrawn and re-sheathed rather than adjusted in situ to reduce embolic risk. Bail-out options, such as chimney grafting via a safety wire in the BCA, remain available. Avoiding excessive tension on the pull-through wire is also crucial to minimize cerebral complications.

RTAD, although rare, carries high mortality and is strongly associated with oversizing.^6^ To mitigate this risk, stent sizing must be carefully tailored; in our case, oversizing was 6.25%. In addition, an ascending aortic diameter ≤40 mm and a proximal landing zone ≥2 cm were applied as anatomical criteria to reduce RTAD^7^ and endoleak risk.

Endoleaks require long-term surveillance. Type IV leaks observed intraoperatively often resolve spontaneously; however, if persistent beyond 6 months, a gap between the BCS and fenestration should be suspected and balloon dilation considered. In-stent thrombosis was managed with continuation of prior single antiplatelet therapy. Mycotic pseudoaneurysm has traditionally been regarded as high risk for endovascular repair, but acceptable outcomes have been reported in infection-controlled patients.^8^ Nevertheless, reinfection remains a concern, necessitating vigilant follow-up.

### Limitations

This report describes a single case, limiting generalizability. The technique is anatomically constrained: supra-aortic branches should be ≤12 mm in diameter, as vessels ≥13 mm require retrograde cervical or subclavian access. Although balloon-expandable stents might theoretically allow treatment of larger vessels, we avoid them because of the risk of deformation during subsequent interventions. Finally, the risk of serious complications—including stroke, RTAD, endoleaks, and in-stent thrombosis—highlights the need for cautious patient selection, lifelong surveillance, and further validation in larger studies.

## CONCLUSION

Zone 0 TEVAR using a triple-fenestrated PMEG with transfemoral bridging stent delivery is technically feasible and avoids cervical delivery routes. This approach may help reduce cerebral embolic risk and represents a promising minimally invasive option for high-risk patients with arch pathology unsuitable for open surgery.

## Supplementary Material

ivaf209_Supplementary_Data

## Data Availability

The data supporting this report are not publicly available due to patient privacy considerations but may be obtained from the corresponding author upon reasonable request.
