# Supplementary material for: Transfemoral Bridging Stent-Graft Delivery in Zone 0 Endovascular Arch Repair With Triple-Fenestrated Endograft
Source: Interdiscip Cardiovasc Thorac Surg. 2025 Sep 16;40(9):ivaf209. doi: 10.1093/icvts/ivaf209 (PMC12548036; doi:10.1093/icvts/ivaf209)
Supplement: ivaf209_Supplementary_Data [file ivaf209_Supplementary_Data.zip › Supplemental material legends.docx]

**Supplemental material legends**

Supplement 1

Computed tomography (CT) findings of coronary artery pseudoaneurysm

A, The initial CT showed a pseudoaneurysm of the left anterior descending artery (arrow). B, A CT scan taken three months after the initial consultation showed that the pseudoaneurysm of the left anterior descending artery had almost disappeared (arrow).

Supplement 2

Follow-up computed tomography (CT) at 1 year likewise demonstrated no endoleaks and a reduction in aneurysm size.
