# Supplementary figures and images for: Transfemoral Bridging Stent-Graft Delivery in Zone 0 Endovascular Arch Repair With Triple-Fenestrated Endograft
Source: Interdiscip Cardiovasc Thorac Surg. 2025 Sep 16;40(9):ivaf209. doi: 10.1093/icvts/ivaf209 (PMC12548036; doi:10.1093/icvts/ivaf209)

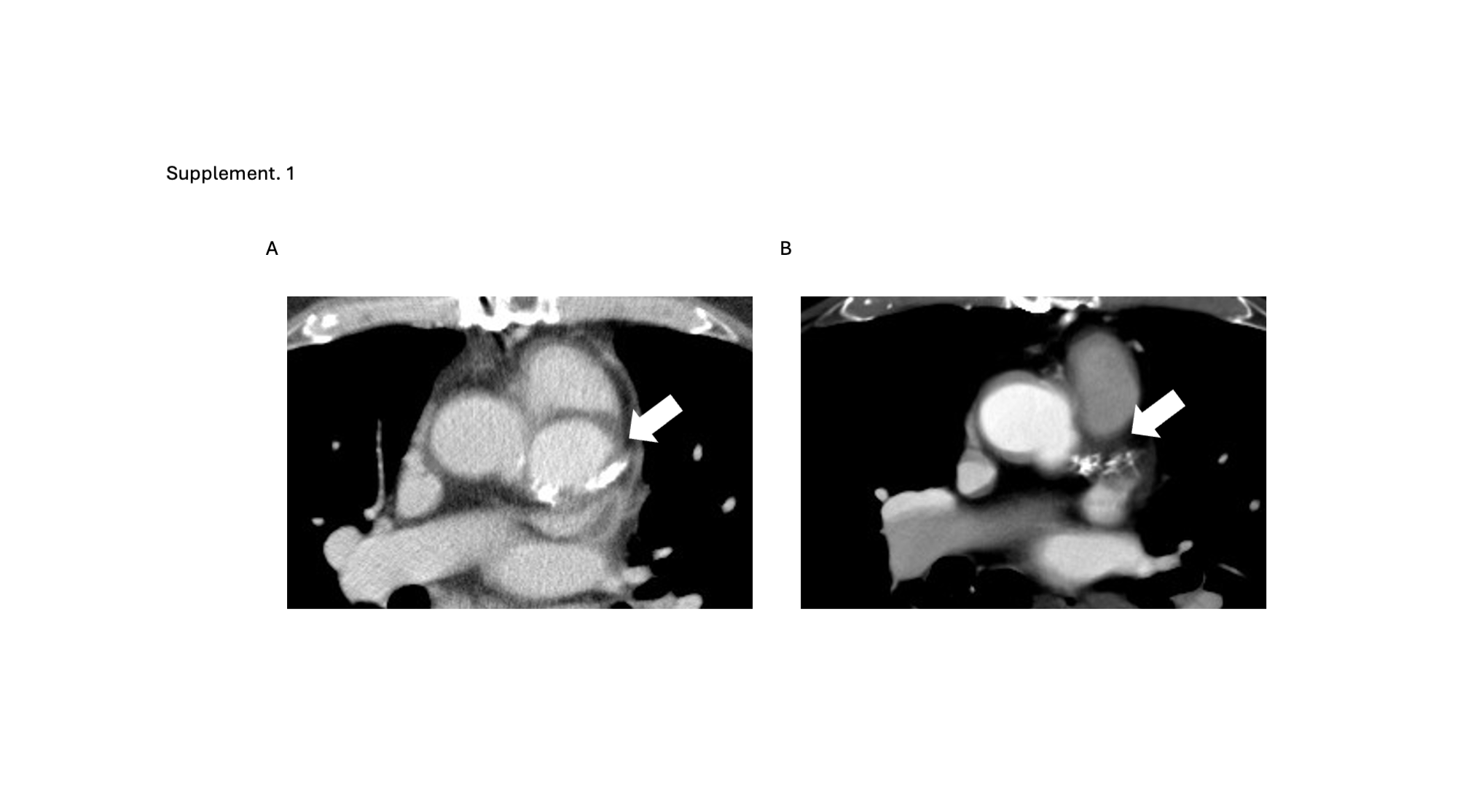

Supplement: ivaf209_Supplementary_Data [file ivaf209_Supplementary_Data.zip › supplement. 1.tiff]

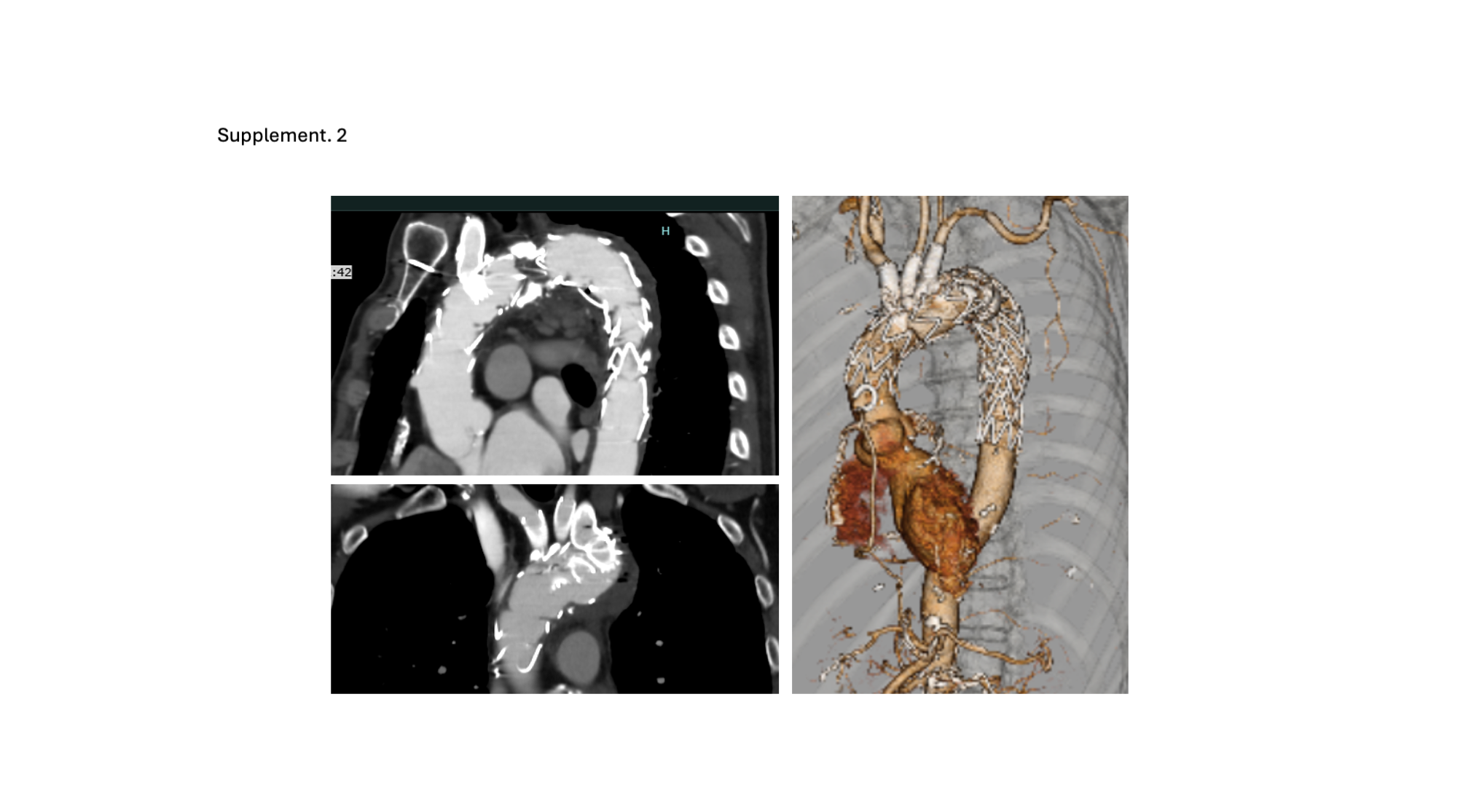

Supplement: ivaf209_Supplementary_Data [file ivaf209_Supplementary_Data.zip › supplement. 2.tiff]
